# Design and Evaluation of a Rapid Monolithic Manufacturing Technique for a Novel Vision-Based Tactile Sensor: C-Sight

**DOI:** 10.3390/s24144603

**Published:** 2024-07-16

**Authors:** Wen Fan, Haoran Li, Yifan Xing, Dandan Zhang

**Affiliations:** 1 Department of Bioengineering, Imperial-X Initiative, Imperial College London, London SW7 2AZ, UK; wf24@ic.ac.uk; 2 Bristol Robotics Laboratory, School of Engineering Mathematics and Technology, University of Bristol, Bristol BS8 1QU, UK; haoran.li@bristol.ac.uk; 3 Visual Information Laboratory, School of Computer Science, University of Bristol, Bristol BS8 1QU, UK; yifan.xing@bristol.ac.uk

**Keywords:** vision-based tactile sensor, monolithic 3D printing, contact reconstruction

## Abstract

Tactile sensing has become indispensable for contact-rich dynamic robotic manipulation tasks. It provides robots with a better understanding of the physical environment, which is a vital supplement to robotic vision perception. Compared with other existing tactile sensors, vision-based tactile sensors (VBTSs) stand out for augmenting the tactile perception capabilities of robotic systems, owing to superior spatial resolution and cost-effectiveness. Despite their advantages, VBTS production faces challenges due to the lack of standardised manufacturing techniques and heavy reliance on manual labour. This limitation impedes scalability and widespread adoption. This paper introduces a rapid monolithic manufacturing technique and evaluates its performance quantitatively. We further develop and assess C-Sight, a novel VBTS sensor manufactured using this technique, focusing on its tactile reconstruction capabilities. Experimental results demonstrate that the monolithic manufacturing technique enhances VBTS production efficiency significantly. Also, the fabricated C-Sight sensor exhibits its reliable tactile perception and reconstruction capabilities, proofing the validity and feasibility of the monolithic manufacturing method.

## 1. Introduction

Robotic manipulation tasks frequently involve multiple physical contacts between the robots’ end-effector and the external environment, where tactile sensing provides crucial capabilities for perceiving these interactions [1]. Unlike vision sensors, tactile sensors excel in fine texture identification and force/shear mapping in the local contact area, focusing on capturing the dynamic information generated by the contact process rather than the global 3D environmental sensing. Tactile sensing plays a pivotal role in human-robot interaction and precise in-hand manipulation tasks. Various tactile sensors employing different mechanisms, such as magnetic sensors [2,3] is missing, please check and revise it., capacitive sensors [4,5], resistive sensors [6,7], vision-based tactile sensors (VBTSs) [8,9,10,11,12], etc. Among these types of sensors, VBTSs are distinguished by various advantages. Assisted by an embedded camera, the high-resolution tactile data can be captured and transferred into standard image format naturally during imaging instead of the time-consuming analog-to-digital conversion post-process and complex integration circuit design for data collection. This property leads to the evident benefit of high spatial resolution, such as GelSight [8], which can accurately reconstruct fine textures such as fingerprints or clothing fiber. VBTSs, with their image-based output, enable the application of advanced machine-learning models for tactile perception. For instance, the contact geometry and relative pose are able to be estimated by Graph Neural Networks with other Voronoi-based augmented features [13], while contact depth and force can be predicted through pixel intensity regression and deep learning [14]. Furthermore, integrating vision and tactile modalities facilitates comprehensive multimodal perception, achieved through mechanisms like see-through-skin technology [12,15].

Despite their promising capabilities, the development of VBTSs is hindered by current manual manufacturing techniques. This traditional fabrication method is labour-intensive and variable, leading to potential inconsistencies in sensor quality and performance. Moreover, the diverse designs documented in various studies complicate the standardization of VBTS production and pose significant challenges in adapting internal structures and external dimensions for different robotic tasks. Typically, most existing VBTSs comprise three main components: the contact module, perception module, and illumination module [11,12,16]. The manufacturing process, particularly within the contact module, involves intricate steps such as skin application, coating deposition, marker integration, elastomer casting, lens assembly, and base mounting. For instance, producing a single-layer elastomer via the prevalent mould-forming method entails multiple critical steps: (1) mould design and fabrication, (2) solution preparation, (3) degassing, (4) elastomer casting, (5) thermal curing, and (6) de-moulding, which necessitate specialised equipment including vacuum extractors, thermal ovens, airbrushes, and laser cutters. However, the complexity even escalates for VBTS designs featuring multi-layer structures, intricate marker patterns, or delicate coatings.

In addition to complex processes, the reliance on manual skills for VBTS manufacturing and assembly poses another significant challenge: the quality of the final product, including assembly precision and error rates, heavily relies on the proficiency and experience of the workforce. This variability complicates the mastery and replication of VBTS designs by researchers, impacting their consistency and scalability. These errors contribute to manufacturing flaws in individual components and result in inconsistencies across production batches, leading to varying outputs among identical VBTS units operating under similar conditions. Also, such hardware inconsistencies pose substantial challenges for developing generalised and adaptable software solutions, particularly signal processing and calibration algorithms, which are critical for deep learning models that rely extensively on data collected from these sensors [17]. Moreover, as per [16], VBTS manufacturing costs encompass processing, material, equipment expenses, etc. The intricate procedures, specialised equipment requirements, and skilled labour involved in existing VBTS manufacturing methods significantly escalate costs. Additionally, the sequential assembly method and lack of specialised assembly equipment contribute to prolonged assembly times, even when utilizing prefabricated components.

In summary, the challenges of low assembly efficiency, high error rates, and performance variability among VBTS units represent significant obstacles to their reliability and standardisation. These issues not only impede prototype development but also hinder the mass production of VBTSs. Consequently, current manufacturing techniques often yield unsatisfactory results in terms of production quality, particularly in large-scale production and maintaining consistency. For a comprehensive analysis, we examine the challenges faced by current VBTS manufacturing through the lens of four primary complexities: design, process, time, and quality. Each of these aspects is explored in detail to provide a sequential understanding of the issues involved.

### 1.1. Design Complexity

Changes in the structural design of VBTS, such as the addition or modification of components, can significantly impact their manufacturing process. Conversely, the specific requirements imposed by current manufacturing methods constrain the design possibilities of VBTSs in return. This mutual limitation complicates the overall VBTS manufacturing process.

- Large Category Difference: Different tactile sensing mechanisms rely on distinct hardware structures. For example, GelSight-type sensors [8,18,19] require reflective coatings, whereas marker-based sensors [9,11,20] rely on the marker patterns. The lack of a standardised manufacturing process results in significant disparities in the production of various categories of VBTSs.
- Low Customised Flexibility: The traditional manufacturing methods are usually monofunctional and lack flexibility. For example, the mould-forming method is widely used in elastomer manufacturing for VBTSs. However, modifying the original design of such VBTSs requires additional time for remanufacturing the new mould. This issue, often referred to as the ‘mould dependency’ problem [21], severely restricts design flexibility.

### 1.2. Process Complexity

VBTS manufacturing consists of two primary steps: first, the fabrication of subcomponents, followed by the final assembly process. However, manufacturing and assembly may interact in a mutually influential manner.

- Cumbersome Manufacturing Procedure: The preparation of a single-layer elastomer using the mould-forming method includes multiple steps: mould manufacture, solution preparation, air elimination, mould casting, heat curing, and mould release. Additional procedures, such as dyeing and stiffness adjustment, may also be required, making the procedure more cumbersome.
- Complicated Equipment: With the increase in manufacturing steps, additional specialised equipment becomes necessary. Typically, these devices serve a single manufacturing procedure, posing a significant burden on small or individual research teams.
- Special Manual Skill: Certain manual processes necessitate experience and specialised skills, making them challenging for beginners or other researchers to master. Consequently, this limitation hampers the design sustainability and restricts horizontal collaboration across different research groups.

### 1.3. Time Complexity

Most VBTSs have extended production lead times, negatively impacting the iteration speed of proof-of-principle prototypes as well as the scale-up manufacturing of mature products. It is often overlooked that increased time complexity also elevates the overall manufacturing costs.

- Long Manufacturing Time: The manufacturing of elastomers involves significant time for preparing, casting, and curing the silicone solution, typically ranging from hours to several days. Similarly, painting coating layers and casting lenses with complex shapes also demands extended time.
- Low Assembly Efficiency: Due to the serial assembly workflow, achieving direct assembly of the final product using all pre-fabricated subparts is challenging. Additionally, the lack of assembly equipment further reduces overall efficiency.

### 1.4. Quality Complexity

With existing manufacturing techniques, it is difficult to manufacture VBTSs in bulk with consistent quality.

- Large Assembly Errors: Most VBTS manufacturing involves manual assembly, resulting in random assembly errors, which magnify the adverse effects of any existing manufacturing errors.
- Large Individual Variability: Assembly errors can cause output data variations among VBTSs with identical designs. This complicates the generalisation of subsequent signal processing and calibration algorithms, especially for deep learning models.

**Figure 1:**
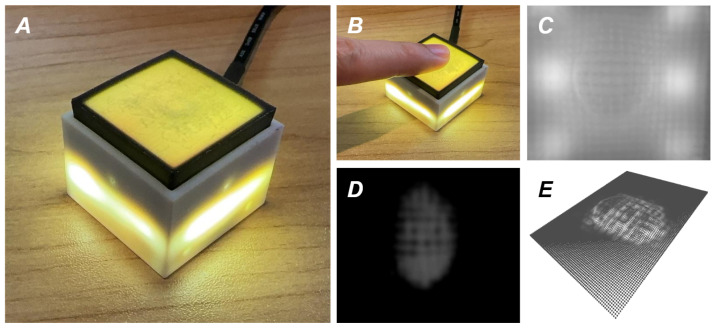
(**A**) Overall view of the assembled C-Sight sensor, where the proposed rapid monolithic manufacturing technique fabricates the whole contact module and the camera base; (**B**) We use finger to touch the C-Sight skin; (**C**) Image directly captured by C-Sight without processing; (**D**) Depth estimation result; (**E**) Contact reconstruction result.

To address these challenges, this paper introduces a unified monolithic 3D printing technique designed for the rapid manufacturing of VBTSs. Inspired by the impressive flexibility of the integrated printing proposed in [21], embedded grid structures can serve as an alternative option to silicone elastomer, easily adapted to various VBTS designs. The novel monolithic manufacturing technique leverages advancements in additive manufacturing to create sensors with enhanced precision and functionality. By simplifying the manufacturing process into a single, unified printing sequence, this method not only accelerates production but also ensures greater consistency and integration of the sensory components. To demonstrate the efficiency of such a proposed method, we create a C-Sight sensor using monolithic manufacturing as displayed in Figure 1A, which gains inspiration from DTac [10,14], but with distinct advantages in rapid manufacturing. Furthermore, to validate the feasibility, versatility, and practicality of our rapid manufacturing approach, we conduct a comprehensive quantitative evaluation.

The **main contributions** of this paper are outlined as follows:

- We introduce a unified manufacturing framework for VBTS, employing monolithic manufacturing to streamline production and reduce associated costs.
- We develop a novel VBTS sensor, C-Sight, leveraging monolithic manufacturing to demonstrate the adaptability and efficacy of our fabrication method.

## 2. Design and Fabrication

### 2.1. Comparison between Typical 3D-Printing Methods

As discussed in the previous section, the primary challenge in manufacturing VBTS lies mostly in the contact module, with recent research focusing on enhancing its fabrication techniques [9,11,16,21,22]. Notably, 3D printing technology has demonstrated significant potential in this regard. For example, TacTip [9] and ChromaTouch [22] utilised 3D-printing technology to fabricate their bases, markers, and skins, while MagicTac [21] even fabricated its whole contact module including the elastomer and lens. The specific 3D-printing machines used in their manufacturing are Stratasys J826 (https://www.stratasys.com/en/3d-printers/printer-catalog/polyjet/j8-series-printers/j826-prime-3d-printer/, accessed on 9 July 2024) and J735 (https://support.stratasys.com/en/Printers/PolyJet-Legacy/J735-J750, accessed on 9 July 2024), both of which utilise PolyJet Printing (PP) [23], a superior printing technology with high-resolution performance on multi-material additive fabrication. A comparison between PP and other typical 3D-printing methods is shown in Table 1. Fused Deposition Modeling (FDM) [24] has the lowest printing costs but the worst printing quality, and it is also hard to utilise flexible materials except for the opaque thermoplastic polyurethane (TPU). Stereolithography (SLA) [25] offers the highest printing quality, but it lacks multi-material printing capabilities. Additionally, its post-processing includes the cleaning of liquid resins, requiring specialised dissolving equipment. Selective Laser Sintering (SLS) [26] offers ease of post-processing due to the absence of supports; however, it still lacks the ability to print multi-material components for transparent or flexible materials. In comparison, PP stands out by combining the advantages of the aforementioned techniques with fine printing accuracy, flexible multi-material function, and straightforward post-process, only with its primary limitation being the relatively high cost of the printing equipment.

### 2.2. Monolithic Manufacturing for VBTS

Based on the PP, we propose a monolithic manufacturing technology for the VBTS contact module, which simplifies the production process as follows. Initially, the printing process commences with a thin layer of support material on the printer’s tray, composed of a soft, translucent gel. This layer ensures the horizontal stability of printed objects with non-flat bottom shapes and facilitates harmless separation from the print tray upon completion. Then, the components are printed layer by layer according to the CAD model of the VBTS design. A typical fabricating sequence often includes the skin, coating, markers, elastomer, lens, and base. Once the printing process concludes, the printed object is detached from the printer’s tray, and then the bottom support part is removed. This removal is facilitated by the material’s soft texture and thin thickness, allowing for easy separation using water spray or hand tools within a few minutes. After completing the above steps, the entire fabrication process of the VBTS contact module is finished, including the manufacturing and assembly. Finally, the contact module can be directly assembled with the illumination and camera modules to form the complete VBTS system without any extra process. Therefore, the whole process above is defined as monolithic manufacturing technology.

The printing materials commonly used in the PP process are listed in Table 2. According to their attributes, Vero (https://www.stratasys.com/en/materials/materials-catalog/polyjet-materials/vero/, accessed on 9 July 2024) is suitable to manufacture the base, lens, and 2D marker [27] due to its resin-like properties and full-color capability. Similar to Tango (https://www.stratasys.com/en/materials/materials-catalog/polyjet-materials/tango/, accessed on 9 July 2024), Agilus30 (https://www.stratasys.com/en/materials/materials-catalog/polyjet-materials/agilus30/, accessed on 9 July 2024) is a rubber-like flexible material. Its colour and stiffness can be adjusted by mixing with Vero material, making it suitable for skin, elastomer, and flexible marker applications. The support material is gel-like with a translucent appearance. Its ultra-soft property allows for effective utilization in designs that structurally combine it with other materials, including Vero. For the base of VBTS, DraftGrey (https://www.stratasys.com/en/materials/materials-catalog/polyjet-materials/draftgrey/, accessed on 9 July 2024) and Digital ABS (https://www.stratasys.com/en/materials/materials-catalog/polyjet-materials/digital-abs-plus/, accessed on 9 July 2024) are considered ideal substitutes for Vero material. As demonstrated by the existing research [9], three sub-components of VBTS, including the base, marker, and skin, can be effectively manufactured using 3D printing. However, its feasibility for creating the other three remaining components, such as the lens, elastomer, and coating, is rarely discussed.

#### 2.2.1. Lens

According to [21], the lens can be manufactured properly using VeroClear, which could be regarded as the alternative option for acrylic. Alternatively, VeroUltraClear provides higher light transmission (95%) and clarity than VeroClear.

#### 2.2.2. Elastomer

Agilus30 Clear is a promising material for elastomer manufacturing because of its flexibility and transparency. However, its stiffness limitation, with a minimum hardness of 30A, may not be suitable for softer elastomer requirements in many VBTS designs. As per [21], the stiffness of printed elastomer could be further reduced by introducing the multi-layer grid structure.

#### 2.2.3. Coating

Both reflective and controllable coating require metal material such as flake/powder pigments or foil. However, PP has not supplied the printing materials that contain metal pigment until now. Given the materials listed in Table 2, the conventional coating is difficult to realise through monolithic manufacturing at the current stage.

### 2.3. Comparison with Current VBTS Manufacturing

**Figure 2:**
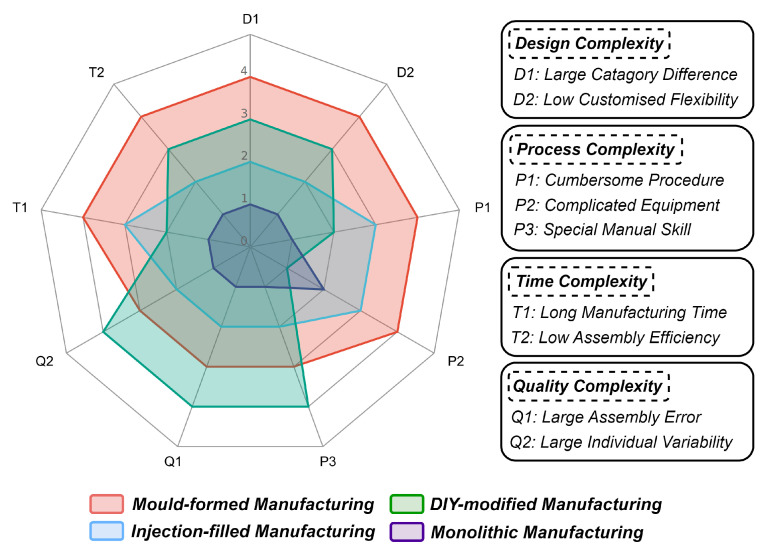
Comparison of different manufacturing methods for VBTS. The manufacturing methods can be classified into four categories based on the method by which the elastomer is made, namely (1) Mould-formed; (2) Injection-filled; (3) DIY-modified; and (4) Monolithic manufacturing. We qualitatively compare their manufacturing by ranking them according to metrics including design, process, time, and quality.

In general, the traditional manufacturing technique for VBTS can be categorised into four categories based on elastomer preparation methods, which are **mould-formed** manufacturing, **injection-filled** manufacturing, **DIY-modified** manufacturing, and also the **monolithic** manufacturing which is proposed in this work. Due to the lack of quantitative metrics, these four methods are evaluated qualitatively based on the aforementioned complexities defined in the Introduction section, including design, process, time, and quality in four parts and nine related metrics. Scores ranging from one to four are assigned to rank each method from best to worst based on these metrics. Detailed results of this evaluation are presented in Figure 2.

Based on the results, mould-formed manufacturing ranks lowest due to its limited customization flexibility, complex fabrication steps, and extended production duration. Conversely, DIY-modified manufacturing excels in reducing equipment dependency, but it faces significant limitations in enhancing production quality and stability. Injection-filled manufacturing, which employs 3D-printing technology as a partial replacement for mould casting, can be regarded as an advanced iteration of mould-formed manufacturing. However, it remains limited by the time-intensive nature of the silicone preparation and gel injection process. In comparison, monolithic manufacturing offers a more effective solution to the constraints inherent in current VBTS manufacturing processes. It holds the potential to evolve into a universal design and manufacturing framework for VBTS, thereby enhancing design flexibility, streamlining production, and lowering overall costs. To illustrate this efficiency, the subsequent sections provide a detailed description of the design and manufacturing process of the C-Sight, created by the proposed monolithic manufacturing.

### 2.4. Design and Fabrication of C-Sight

Here, we introduce the novel C-Sight sensor fabricated using the proposed monolithic manufacturing technique, which is inspired by the structure of the DTac-type sensor [10,14]. DTac distinguishes itself from other tactile sensors that rely on reflective coating or marker patterns due to its novel combination of outer opaque black layer, middle translucent elastomer layer, and internal transparent elastomer layer, providing impressive tactile feature mapping ability. However, such a stacked architecture places a challenge in mould-formed manufacturing, where three different silicone formulations with varying compositions must be manually cast layer by layer. Referring to Figure 2, such a manufacturing method may lead to significant design, process, time, and quality complexities. In this case, we aim to fabricate a DTac-type sensor, C-Sight, to demonstrate that the proposed monolithic manufacturing method has the feasible ability to create VBTS with complicated structures.

**Figure 3:**
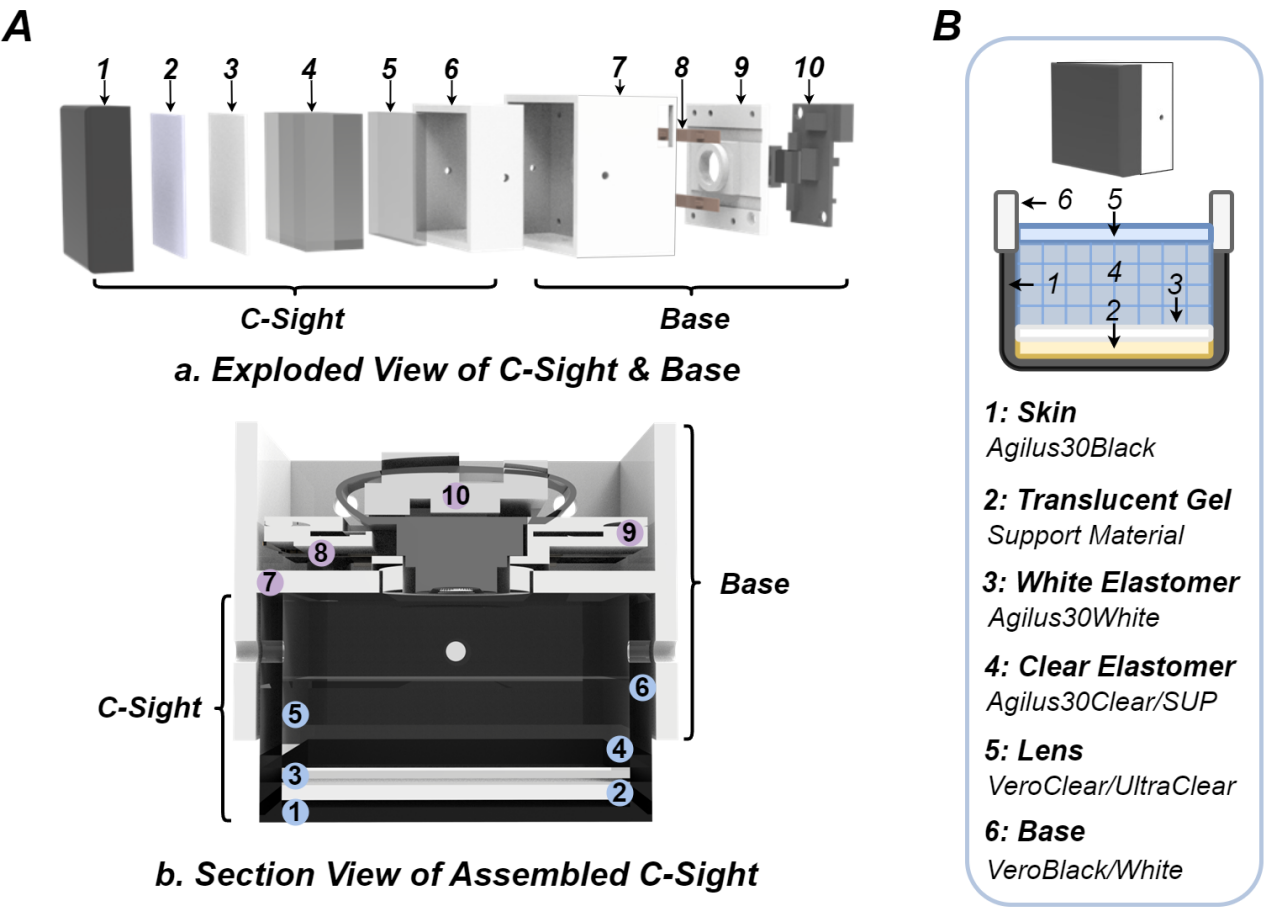
Overview of the C-Sight sensor design. (**A**) Exploded view of C-Sight and newly designed base, including the illustrations of sub-parts: (1) opaque skin made of Agilus30 Black, (2) translucent layer made of support material, (3) white layer made of Agilus30 White, (4) transparent layer made of the multi-layer grid, (5) lens made of VeroClear, (6/7/9) base made of VeroWhite, (8) LEDs, (10) camera. (**B**) Section view of assembled C-Sight. C: The design diagram and specific printing materials assigned for each subcomponent.

The exploded view and section view of C-Sight are illustrated in Figure 3. Between the external opaque skin and the internal transparent elastomer, two additional components are present: a translucent layer composed of support material and a pure white layer fabricated from Agilus30White. Similar to the DTac sensor’s mechanism, the deformation resulting from an object’s contact induces a variation in the distance between C-Sight’s outer black skin and embedded white skin. This deformation is facilitated by the ultra-soft and translucent properties of the support material. Theoretically, from the camera’s imaging, places, where the distance between black and white skins is shortened (concave inwards), appear darker in pixels. As shown in Figure 1D, this unique feature of C-Sight is the subtraction between the reference and camera imaging. This property makes it possible to infer the tactile texture and depth information from the pixel values.

As displayed in the exploded and section views of the sensor assembly in Figure 3A, the C-Sight contact module comprises six components: opaque skin (1), a translucent layer (2), a white layer (3), a transparent layer (4), a lens (5), and the mounting base (6). Also, the designed base consists of four parts, including a home base (7), LED lights (8), a camera base (9), and a camera (10). C-Sight uses six white LEDs to provide illumination in the vertical direction where the cross-sectional view of the sensor base reveals that the light emitted by the LEDs is scattered through the intermediate baffles in the home base. Figure 3B presents a schematic diagram detailing the materials used for each subpart of the C-Sight contact module. Compared to the original design of the DTac, it can be noted that C-Sight introduces an extra structure, a white layer (3) between the translucent layer (2) and the transparent layer (4). The role of this white layer is to better filter the tactile image background and enhance the contrast of the black layer when contact occurs. It’s important to note that this work does not aim to prescribe a standardised or definitive design for C-Sight to match or surpass the tactile sensing performance of the official DTac sensor. Instead, the details of its internal structure, each layer’s dimensions, and the materials’ distribution can be modified flexibly, depending on specific application requirements. This approach aims to demonstrate the potential that if a DTac-type sensor can be produced through monolithic manufacturing, then VBTSs with other complex structures can also be feasibly developed, offering developers a solution to realize diverse and novel designs. Therefore, C-Sight serves more as a template, encouraging researchers to explore their own innovative VBTS structures using monolithic manufacturing techniques.

## 3. Performance Evaluation

In this section, we designed several experiments to quantitatively evaluate the effectiveness of the proposed monolithic manufacturing technique and the performance of the C-Sight sensor.

### 3.1. Evaluation of Monolithic Manufacturing Technology

Given the manufacturing limitations of the current VBTSs, the proposed monolithic manufacturing offers a potential solution to address the challenges in design, process, time, and quality complexities. In addition to post-processing steps such as support removal and one-off purchase costs of equipment, the primary expenses of monolithic manufacturing in practical applications are predominantly due to printing time and material consumption costs. These factors are further evaluated below on test samples using three distinct parameters: print material, size, and capacity.

#### 3.1.1. Print Material

The typical printing materials in PP are summarised in Table 2. Considering that the J826 printer has eight print heads and one is dedicated to the support material (SUP706), a total of seven other materials can thereby be utilised. As shown in Table 3, we selected a range of printing materials, including Agilus30Black (AB), Agilus30White (AW), Agilus30Clear (AC), DraftGrey (DG), VeroBlackPlus (VB), VeroPureWhite (VW), and VeroClear (VC), which can meet the requirements the design and manufacturing requirements of most VBTSs. Excluding 20%VAT, their unit prices are, respectively, 0.2 £/g for the Agilus30 series, 0.1 6£/g for the Vero series, 0.086 £/g for DraftGrey, and 0.02 £/g for SUP706. The evaluation was performed on GradCAD (https://support.stratasys.com/en/Resources/Software-Download, accessed on 9 July 2024), the official software provided by Stratasys. To ensure the accuracy of simulations for all test samples, specific printing parameters were set: finish level to ‘Glossy’, support strength to ‘Standard’, and print mode to ‘High Mix’.

**Figure 4:**
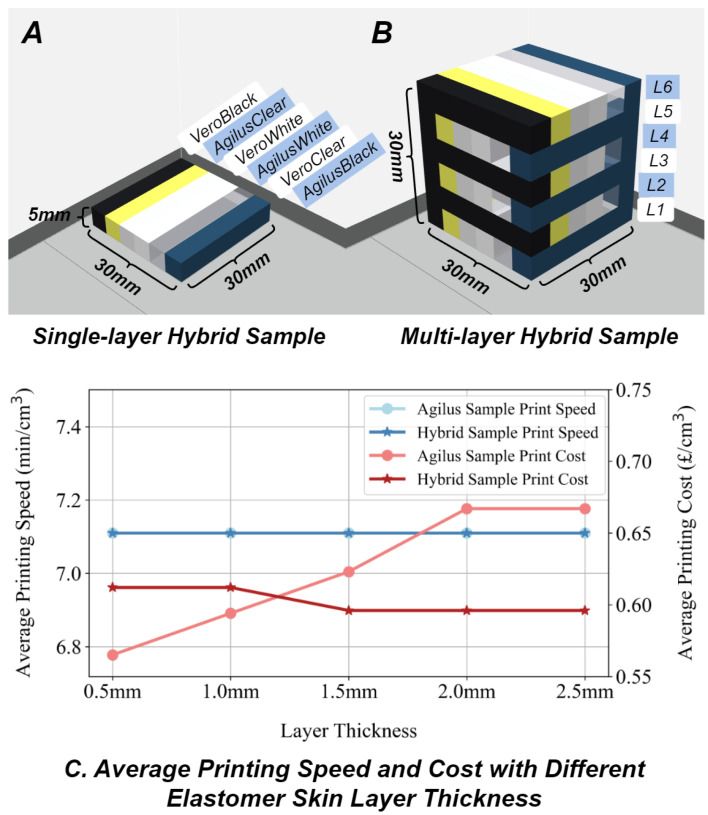
(**A**) A single-layer sample is introduced for manufacturing cost evaluation, consisting of six blocks that can be made from hybrid materials. (**B**) Another multi-layer sample with up to six floors is used to simulate the monolithic manufacturing situation of VBTSs with complex structures. (**C**) The skin layer thickness of multi-layer grid elastomer does not influence printing time but the cost when the original sample is made of pure Agilus30 printing material.

As illustrated in Figure 4A, a single-layer sample was designed with an overall size of 30 mm × 30 mm × 5 mm. It consists of six blocks, each with dimensions of 5 mm × 30 mm × 5 mm. Following the configuration with six printing materials, including the Agilus30 and Vero series, the manufacturing costs in terms of time and price are summarised in Table 3. It can be concluded that despite only six being specified for use, eight loaded materials, including DraftGrey and SUP706, are all consumed, the difference being in the exact volume. Similarly, although a specific material is designated for printing, such as a single Agilus30Black, all loaded materials are consumed due to the printer’s workflow, with the specified material being predominantly used. Additionally, the average print speed of DraftGrey and Vero materials is approximately 1.33 times faster than that of Agilus30. For objects composed of hybrid materials, the speed is determined by the slowest material in the mixture. Regarding unit material consumption cost, pure Agilus30 is more expensive than Vero, with mixtures of the two presenting intermediate cost levels.

The evaluation results, as shown in Figure 4C, indicate that the layer thickness of multi-layer grid elastomer [21] does not significantly influence the printing speed. For the object entirely made by Agilus30, a thinner layer thickness increases the usage of support material, which is cheaper than Agilus30, thereby decreasing the overall printing consumption cost. In the case of objects made from hybrid materials, however, the impact of this phenomenon is less pronounced.

#### 3.1.2. Print Size

As illustrated in Figure 4B, another multi-layer sample was introduced, comprising six layers of single-layer hybrid samples, arranged in a twisted stack formation, enabling the simulation of the complex structural designs found in actual VBTS. By adjusting the dimensions of the multi-layer hybrid sample, the correlation between the size of the printed object and its manufacturing cost was established, as shown in Table 4. Given the alternating arrangement of Agilus30 and Vero in each single-layer sample, dimensions of 10 mm, 20 mm, and 30 mm were selected for the X/Y axes to mitigate the influence of varying materials. The Z axis was not subject to this material variation, allowing for the selection of heights measuring 5 mm, 10 mm, 15 mm, 20 mm, 25 mm, and 30 mm. The results demonstrate that changes in the dimensions of the printed object along the X, Y, and Z axes—corresponding to length, width, and height—have similar impacts on manufacturing outcomes. Specifically, an increase in any of these dimensions leads to a higher unit printing speed and a lower unit material consumption cost.

#### 3.1.3. Print Capacity

**Figure 5:**
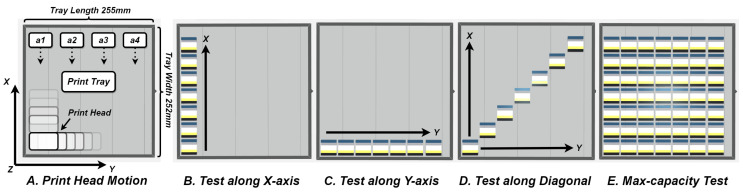
Experiment setups used to evaluate manufacturing speed and cost. (**A**) The print tray of the J826 printer includes about four column areas where the print head usually starts from the first area, a1, and moves along X, Y, and Z axes. (**B**–**D**) Tests for different location arrangements of print samples on the tray whose print time and price are recorded for comparative analysis. (**E**) The maximum capacity within a single print batch includes 49 test samples with a 7 × 7 arrangement.

As shown in Figure 5A, the print head moves along the X, Y, and Z axes during the printing process, confined within the boundaries of the print tray. The planar dimensions of this tray are 255 mm by 252 mm. The available space of the print tray is divided into four areas, a1, a2, a3, and a4. Generally, the printing starts from a1, and each print travels a full stroke in the X direction, while the Y direction depends on the number or size of printed objects. Therefore, we conducted various tests to assess different printing capacities, encompassing the number of print objects and their arrangements, as indicated in Figure 5B–E; results are summarised in Table 5. When printed objects are aligned along the X direction, the unit print speed and unit material consumption cost decrease dramatically, from 7.11 min/cm^3^, 0.596 £/cm^3^ to 1.30 min/cm^3^, 0.375 £/cm^3^, with the enhancements of 81.72% and 37.08%, respectively. In contrast, this pattern is not observed in the Y axis, where the values exhibit only minor fluctuations. This discrepancy can be attributed to the operating principles of the PP printer. Increasing the number of objects in the X axis does not alter the print head’s travel path. However, an increase along the Y axis leads to a more extensive travel path across the a1, a2, a3, and a4 zones, consequently resulting in higher manufacturing costs. This phenomenon is further verified by comparing tests conducted along the diagonal and at maximum capacity. Despite a sevenfold difference in the quantity of printed objects, the total printing duration remains constant for both scenarios. Consequently, this results in disparities of 6.78-fold and 1.78-fold in unit printing speed and material consumption cost, respectively. These findings underscore the importance of optimising batch capacity in monolithic manufacturing processes to achieve significant reductions in both time and cost, particularly for high-volume production scenarios.

In conclusion, the monolithic manufacturing of VBTS does not increase manufacturing costs in comparison with utilising pure Agilus30 or Vero material. The efficiency of monolithic manufacturing improves with the size of each sensor. Moreover, by strategically arranging the printed sensors during mass production, both the unit printing speed and material consumption costs can be significantly reduced.

### 3.2. Evaluation of C-Sight Performance

Based on the proposed monolithic manufacturing technology, a new tactile sensor named C-Sight has been developed, as introduced in Figure 3. This section evaluates the manufacturing process and tactile sensing performance of C-Sight.

#### 3.2.1. Manufacturing Performance

Following a setup similar to that used in the last section, the manufacturing performance of C-Sight was also evaluated in GradCAD, and the related results are summarised in Table 6. Using Figure 3A, B as references, the overall dimensional size of the C-Sight contact module is 26.5 mm × 26.5 mm × 13.5 mm, with the total volume of about 6.5 cm^3^. The thickness values of the outer opaque skin (1), translucent layer (2), white layer (3), multi-layer grid elastomer (4), and lens (5) are 0.5 mm, 1 mm, 0.5 mm, 4 mm, and 2 mm, respectively, which are all freely customised. For single manufacturing, each C-Sight requires 10 g Agilus30 Black/Clear, 10 g VeroClear/White, 3 g GraftGray, and 8 g SUP706, with the printing time/cost of about 1 h/4.4£. This rapid production and low-cost capability are rare compared to the current common VBTSs, which may need days-level production time and several times the manufacturing cost.

**Figure 6:**
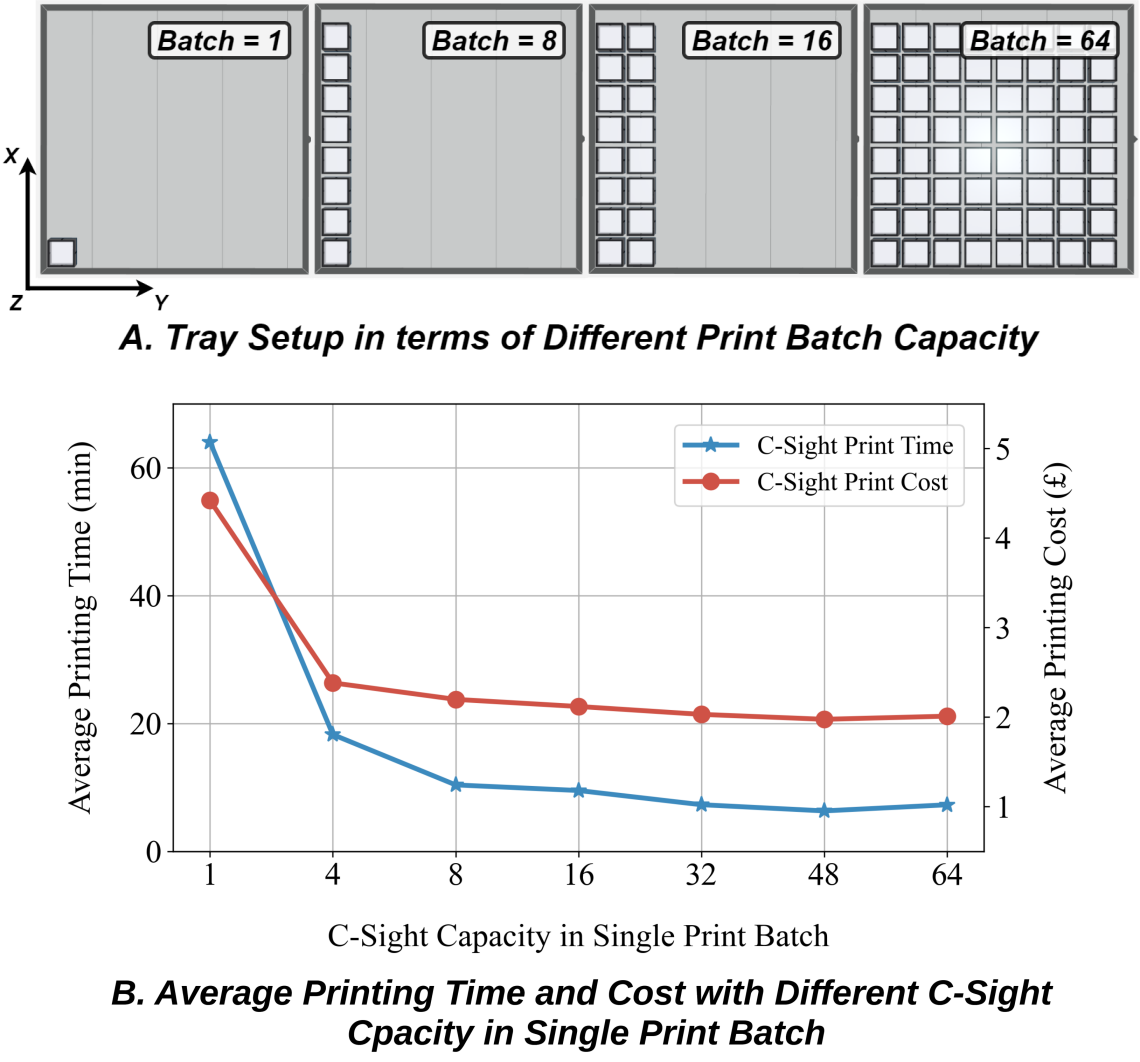
(**A**) The capacity of C-Sight can reach from a minimum of 1 to a maximum of 64 in a single print batch. (**B**) With batch capacity increasing, the average printing time and cost of C-Sight both drop down significantly.

Based on the conclusions drawn in the previous section, we also evaluated the C-Sight on mass production; the setup is displayed in Figure 6A. Fully consistent with the conclusions reached earlier in Table 5, when the printing batch gradually increases from single towards 64, the average manufacturing time and cost decrease from 64 min and GBP 4.418 to 6.31 min and GBP 1.974. This translates to an improvement of 90.14% in time and 55.32% in cost efficiency. Figure 6B illustrates that the optimal point lies roughly in the batch = 8 setting, as at this point C-Sight takes up the entire tray channel, maximising production efficiency.

#### 3.2.2. Tactile Sensing Performance

**Figure 7:**
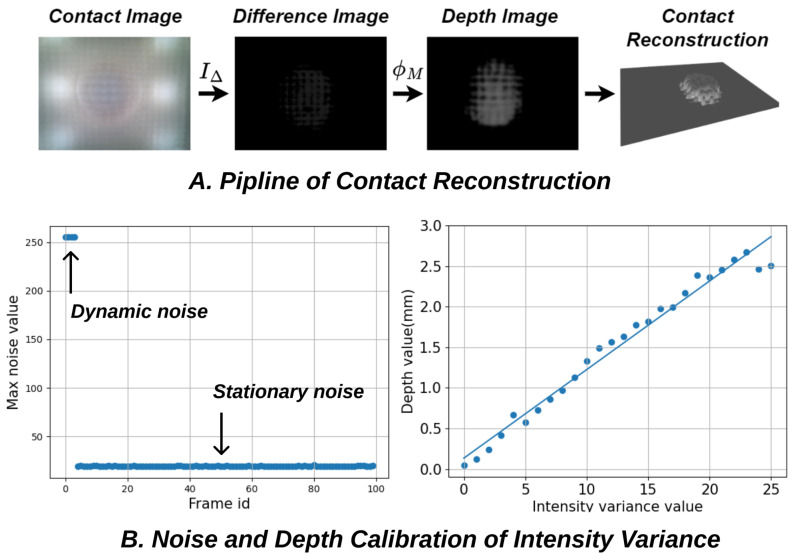
(**A**) The contact image is subtracted by the reference image to generate a different image, which is then mapped towards the depth image through the final contact reconstruction. (**B**) Both intensity variance and depth mapping functions need calibration before contact reconstruction, which is vital to real applications.

To demonstrate tactile sensing capability, C-Sight performs a contact reconstruction task using a methodology similar to that employed by DTac [10], utilising the intensity variance value between sensor frame and reference image to calculate the contact depth. The pipeline of such a reconstruction process is illustrated in Figure 7A.

The core concept of contact reconstruction involves establishing the correspondence between variations in intensity and actual contact depth, a process known as depth calibration. Drawing inspiration from DTac, we compile a mapping list that stores the relationships for collected intensity variance values and then utilises linear regression, denoted as $\phi_{M}$, to model this mapping. This method facilitates the analysis of the collected data and the establishment of a more adaptable relationship. Such steps can be expressed as

(1) $$I_{\Delta }\left(u,v\right)=F_{r}\left(u,v\right)-F_{s}\left(u,v\right)$$

(2) $$D\left(x,y\right)=\phi_{M}\left(I_{\Delta }\left(u,v\right)\right)$$

To achieve noise filtering and depth regression, two additional calibration steps are required before the reconstruction process, as shown in Figure 7B.

- Intensity Variance Calibration: Noise filtering begins with the captured difference image by identifying two types of noise observed in the initial frames, namely dynamic noise and stationary noise, as shown in Figure 7B, left. Dynamic noise occurs in the first few frames due to camera initialization instability, while stationary noise results from fluctuating errors in sensor hardware, including light source flicker frequency, mounting discrepancies in the contact module, and environmental disturbances. Filtering out these system errors, especially stationary noise, is essential. In our test, stationary noise averages around 20, significantly impacting the data. To avoid that, we discard the first 20 frames upon camera activation to address dynamic noise. Then, the maximum noise observed in the subsequent 100 frames is designated as stationary noise and subtracted from each subsequent frame to generate the depth variance image. The intensity variance calibration process described above executes automatically each time the program initialises. Also, to minimise the dynamic fluctuating of stationary noise over time, adding cooling windows at the sensor base reduces heat build-up, and ensures that the thickness of the sensor base or skin is thick enough to avoid random interference from ambient light, which is easily achieved through the proposed monolithic manufacturing method.
- Depth Mapping Calibration: Following the denoising processes described earlier, the intensity-to-depth calibration is performed using Equation (2). In this calibration step, C-Sight is brought into contact with a 6 mm diameter iron ball to establish depth calibration. Given the focus of this paper on verifying the feasibility of C-Sight through monolithic manufacturing, depth calibration is simplified by focusing solely on the center of the sensor surface rather than the entire area. This approach may introduce some distortion at the corners but remains acceptable for functional verification purposes. One example of the depth calibration results is shown in Figure 7B. The mapping regression between depth and intensity variance value is close to the linear relationship, with a depth range of [0,3] mm and an intensity variance value range of [0, 25]. Unlike the automatic process of intensity variance calibration, depth calibration involves manual operation. It is typically performed when C-Sight is initially assembled or when a new contact module is installed. The resulting regression data from the calibration process is stored in a retrievable file format for future use

Compared to DTac, C-Sight utilises printed elastomer instead of pure silicone. Although the embedded multi-layer grid structure brings additional deformation mapping capabilities, it also poses extra challenges, such as the incoherent segmentation of intensity. This issue results in the reconstructed contact surface displaying grid-like depressions, which divide what would otherwise be a smooth area into distinct squares and introduce noise, particularly affecting finer textures. To address such issues, the down-sampling method is introduced to improve the surface smoothness of the contact reconstruction, which is expressed below, where $S_{k}$ represents the down-sampling function with the rate of *k*:

(3) $$I_{\Delta }^{S}\left(u,v\right)=S_{k}\left(I_{\Delta }\left(u,v\right)\right)$$

(4) $$D^{S}\left(x,y\right)=\phi_{M}\left(I_{\Delta }^{S}\left(u,v\right)\right)$$

**Figure 8:**
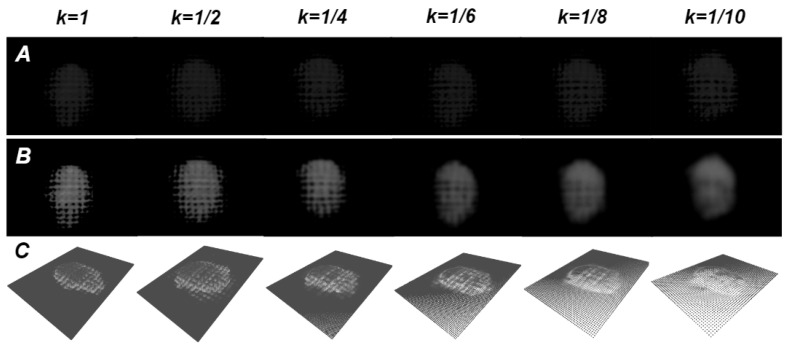
The mapping density of the original difference image (**A**) can influence the smoothness of the depth image (**B**) and contact reconstruction (**C**). The smaller the down-sampling rate, the smoother the downstream results, but the less accurate the details.

The results of down-sampling under different rates are summarised in Figure 8. When *k* is close to 1, the high density of mappings from intensity variance to depth images ensures high accuracy. However, the final reconstruction is significantly influenced by the structure of the multi-layer grid, resulting in a less smooth surface. Conversely, lower values of *k* reduce the mapping density, leading to smoother reconstructed surfaces at the expense of accuracy. Therefore, selecting an appropriate down-sampling rate is crucial to achieve a balanced performance between contact reconstruction accuracy and surface smoothness.

**Figure 9:**
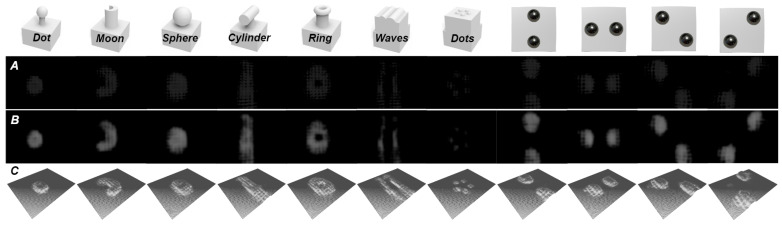
The contact reconstruction examples of different objects with a down-sampling rate, where (**A**–**C**) represent difference image, depth image, and contact reconstruction results separately. From these results, it can be seen that single-point contact, surface contact, multi-point contact, and multi-surface contact can all be reconstructed with a certain spatial resolution.

Following the intensity variance calibration, depth mapping calibration, and down-sampling rate adjustment, C-Sight undergoes contact reconstruction tests using several objects of varying shapes and arrangements, and the experiment results are summarised in Figure 9. The dimensions of test objects include dot (6 mm), moon (7 mm), sphere (10 mm), cylinder (13 mm), ring (8 mm), waves (14 mm), and dots (1.5 mm). It can be seen that C-Sight achieves good shape reconstruction results for point-like and spherical objects, such as dots, spheres, and rings, where a gradient of depth from shallow to deep can be seen from the contact centre to the edge, and even hollow depressions inside the ring can be well perceived. Also, C-Sight achieves a spatial resolution of at least a millimeter scale, capable of distinguishing individual dots in a 5-dot pattern measuring 1.5 mm in size.

However, it is evident that the surface continuity of contact reconstruction for cylindrical objects needs to be improved. Particularly near the edges of the C-Sight contact surface, there are inaccuracies in the reconstructed depth information. Therefore, extra two metal balls with a 4 mm radius are introduced for the uniformity test. The result indicates that the effective reconstruction area covers nearly the central sensing surface, but it should also be noted that the quality of the depth image (Figure 9B) and contact reconstruction (Figure 9C) are influenced by the uniformity of illumination (Figure 7A), as brighter lighting may result in a local saturation of $I_{\Delta }$ shown in Figure 9A.

## 4. Conclusions and Future Work

In this paper, we introduce the C-Sight sensor, fabricated using the proposed monolithic manufacturing technique, which aims to provide a faster, more reliable, and versatile method for the production of VBTSs. The proposed monolithic manufacturing technique represents a significant improvement over traditional VBTS manufacturing techniques by (i) leveraging 3D printing to streamline the production process, enhancing manufacturing efficiency and consistency, and (ii) simplifying the customisation of VBTSs to meet specific requirements across various applications. This technique eliminates the need for a manual fabrication process and significantly reduces production costs and manufacturing time compared to other typical VBTSs. The utility and performance of C-Sight are evaluated in several contact-rich tasks, demonstrating its potential to enhance tactile perception.

Our future research will explore the integration of advanced computational models with monolithically manufactured VBTSs of novel architecture, extending their inference capabilities for tactile sensing. This will pave the way for their use in more complex sensory tasks, such as vision-tactile fusion and robot manipulation. We plan to explore the miniaturisation of sensors for seamless integration with multi-fingered robotic hands. Ultimately, our goal is to establish a standardised, scalable approach for VBTS manufacturing to support the rapid development and deployment of tactile sensing technologies across various domains in robotics.

## Figures and Tables

**Table 1:** Comparison of Typical 3D-printing Technologies (* represents material).

Technology	Layer Height	Surface Finish	Multi-Material	Transparent *	Flexible *	Post Process	Material Cost	Device Cost
FDM [24]	0.05∼0.3 mm	Low	✓	✓	x	Complex	Low	Low
SLA [25]	0.025∼0.1 mm	Extra Fine	x	✓	✓	Complex	High	High
SLS [26]	0.05∼0.2 mm	Rough	x	x	x	Easy	Low	High
PP [23]	∼0.016 mm	Fine	✓	✓	✓	Easy	High	High

**Table 2:** Typical Printing Materials used in PolyJet Printing and Relative Property.

Material	Color	Stiffness	Attribute	Potential
Vero	Clear/Multi-color	Rigid	Resin-like, unlimited full-color tints by mixing	Base/Lens/Marker
Agilus30	Clear/White/Black	Flexible	Rubber-like, adjustable color and stiffness (≥Shore 30A)	Skin/Elastomer/Marker
Tango	Translucent/Gray/Black	Flexible	Rubber-like, adjustable color and stiffness (≥Shore 27A)	Skin/Marker
Support	Translucent	Ultra-soft	Gel-like, with rigid grid, hands-free/mechanical removal	Elastomer
DraftGrey	Multi-color	Rigid	Resin-like, with medium opacity, cheapest and fastest	Base
Digital ABS	Multi-color	Rigid	Resin-like, high temperature resistance and toughness	Base

**Table 3:** Print Cost Summary in terms of Time and Price with Different Material Setup.

Material	AB (g)	AW (g)	AC (g)	DG (g)	VB (g)	VW (g)	VC (g)	Sup (g)	Time (min)	Cost (£) ^1^	T/V (min/cm^3^)	C/V (£/cm^3^) ^1^
AB	8	1	1	2	1	1	1	5	32	3.002	7.11	0.667
AW	1	8	1	2	1	1	1	4	32	2.932	7.11	0.652
AC	1	1	8	2	1	1	1	4	32	2.932	7.11	0.652
DG	1	1	1	8	1	1	1	2	24	1.908	5.33	0.424
VB	1	1	1	1	8	1	1	2	24	2.426	5.33	0.539
VW	1	1	1	1	1	8	1	2	24	2.426	5.33	0.539
VC	1	1	1	1	1	1	8	2	24	2.426	5.33	0.539
Hybrid	2	2	2	2	2	2	2	5	32	2.682	7.11	0.596

^1^ Cost excludes 20%VAT.

**Table 4:** Print Cost Summary with Different Sample Sizes in X/Y/Z.

Size X/Y/Z (mm)	Time (min)	T/V (min/cm^3^)	C/V (£/cm^3^)
10 × 30 ×5	29	19.33	1.137
20 × 30 × 5	31	10.33	0.792
30 × 30 × 5 (X)	32	7.11	0.596
30 × 10 × 5	30	20.0	1.111
30 × 20 × 5	32	10.67	0.722
30 × 30 × 5 (Y)	32	7.11	0.632
30 × 30 × 10	51	5.67	0.573
30 × 30 × 15	69	5.11	0.462
30 × 30 × 20	89	4.94	0.521
30 × 30 × 25	110	4.89	0.514
30 × 30 × 30	128	4.74	0.479

**Table 5:** Print Cost Summary with Different Sample Capacities in X/Y.

Capacity X/Y	Time (min)	T/V (min/cm^3^)	C/V (£/cm^3^)
1 × 1	32	7.11	0.596
2 × 1	33	3.67	0.459
3 × 1	35	2.59	0.454
4 × 1	37	2.06	0.413
5 × 1	39	1.73	0.402
6 × 1	40	1.48	0.392
7 × 1	41	1.30	0.375
1 × 2	70	7.78	0.596
1 × 3	72	5.33	0.546
1 × 4	110	6.11	0.634
1 × 5	110	4.89	0.575
1 × 6	147	5.44	0.575
1 × 7	184	5.84	0.599
7 × 7 Diagonal	233	7.4	0.678
7 × 7 Maximum	233	1.06	0.382

**Table 6:** Manufacturing Cost of C-Sight.

Sensor	Size X/Y/Z (mm)	Volume (cm^3^)	AG (g)	VR (g)	DG (g)	Sup (g)	Time (min)	Cost (£) ^1^	T/V (min/cm^3^)	C/V (£/cm^3^) ^1^
C-Sight	26.5 × 26.5 × 13.5	6.446	10	10	3	8	64	4.418	9.929	0.685

^1^ Cost excludes 20%VAT.

## Data Availability

Please contact authors to obtain data, such as design details of C-Sight.
